# Hierarchical microcrystalline cellulose/SeO_2_–MnO_2_/MWCNTs nanocomposite-based non-enzymatic electrochemical sensor for peroxide analysis in food and milk samples

**DOI:** 10.1039/d6ra07921k

**Published:** 2026-10-08

**Authors:** Sara Z. A. Mahdy, Hend S. Magar, Rabeay Y. A. Hassan

**Affiliations:** a Biosensors Research Lab, Zewail City of Science and Technology 6th October City Giza 12578 Egypt ryounes@zewailcity.edu.eg +201129216152; b Applied Organic Chemistry Department, National Research Centre (NRC) Dokki Giza 12622 Egypt

## Abstract

Reliable determination of hydrogen peroxide (H_2_O_2_) in food matrices requires sensitive and robust non-enzymatic electrochemical sensing strategies. Herein, we developed a hierarchical MCC/MnO_2_–SeO_2_@MWCNTs nanocomposite as a nanostructured-based non-enzymatic electrochemical interface for H_2_O_2_ determination. The proposed architecture integrates microcrystalline cellulose (MCC) with electroactive MnO_2_–SeO_2_ and a conductive multi-walled carbon nanotubes (MWCNTs) network to construct an electrochemically accessible sensing interface. The electrochemically active surface area increased from 0.253 cm^2^ for the bare electrode to 0.412 cm^2^ for the modified electrode, corresponding to a 63% increase. The nanocomposite and its individual components were characterized by Fourier-transform infrared spectroscopy (FTIR), transmission electron microscopy (TEM), cyclic voltammetry (CV), electrochemical impedance spectroscopy (EIS), and chronoamperometry (CA) to define the physical and electrochemcial characteristics. Under optimized conditions, the modified carbon paste electrode exhibited a linear amperometric response to the H_2_O_2_ over 10–1200 µM, with a detection limit of 1.0 µM. The sensor exhibited good selectivity, repeatability, reproducibility, and storage stability. Accordingly, its applicability was tested across yellow cheese, salami, beef, milk, and burger samples, providing recoveries of 95.3–104.61% with low standard deviations (*n* = 5). Therefore, the integration of a MCC matrix with MnO_2_–SeO_2_ and MWCNTs, together with the experimentally demonstrated increase in electrochemically active surface area, provides a practical hierarchical interface for non-enzymatic H_2_O_2_ sensing in diverse food matrices.

## Abstract 2

Schematic representation of a microcrystalline cellulose/SeO_2_-MnO_2_/MWCNTs nanocomposite-modified electrode for direct electrochemical peroxide determination in food and milk samples.

## Graphical abstract

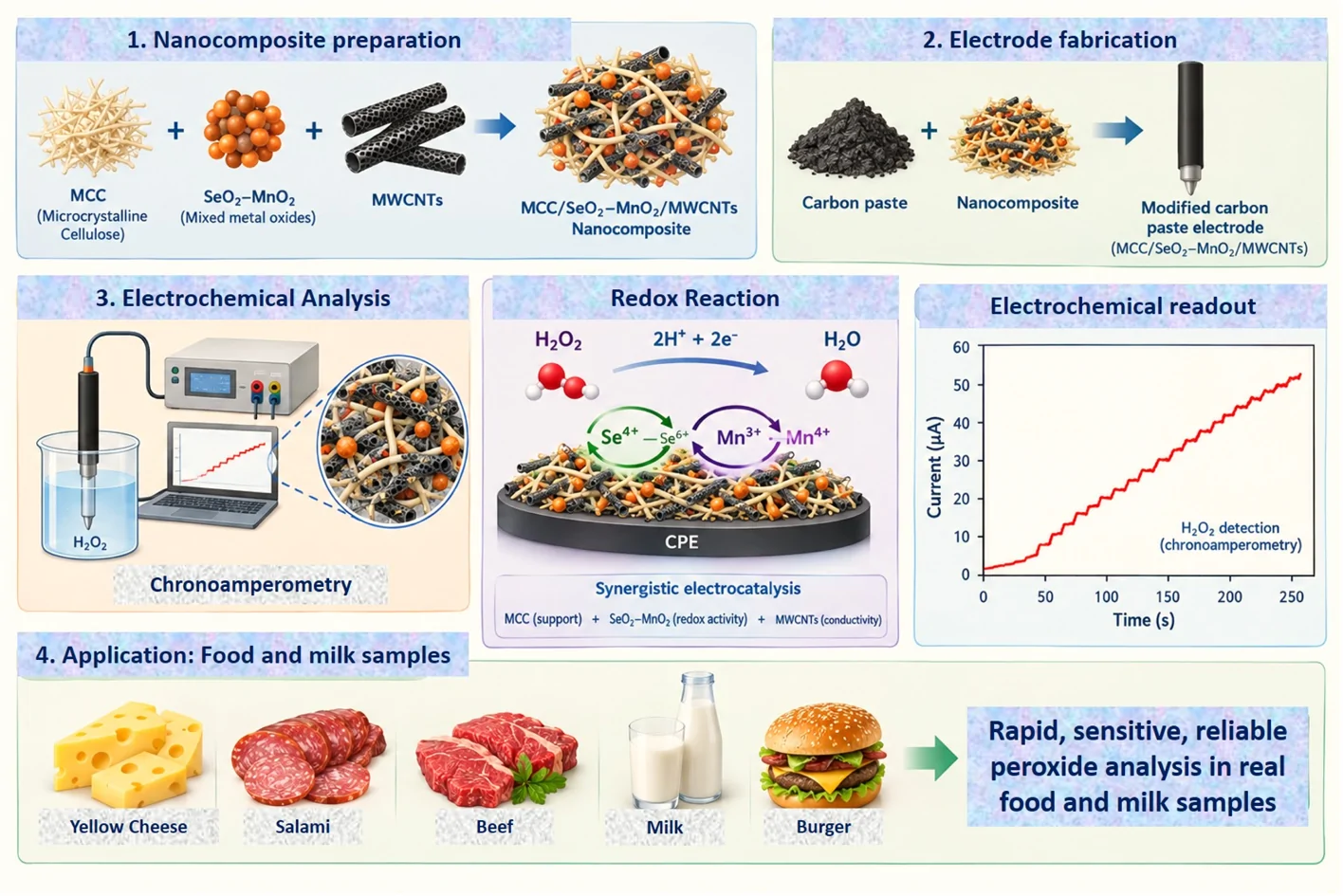

## 1. Introduction

Hydrogen peroxide (H_2_O_2_) is one of the most important reactive oxygen species involved in biological redox processes and is widely used in food processing, pharmaceutical manufacturing, textile and paper industries, wastewater treatment, and disinfection.^1^ At controlled physiological concentrations, H_2_O_2_ participates in cellular signaling and redox homeostasis; however, its excessive accumulation can disturb redox balance and promote sever oxidative damage to proteins, lipids, and nucleic acids.^2^ In food-related applications, H_2_O_2_ is associated with processing, preservation, and quality-control practices, making its reliable determination important for food safety and quality assessment.^3^

Thus, a variety of analytical methods have been developed for H_2_O_2_ determination, including titrimetry, spectrophotometry, fluorescence, chemiluminescence, and chromatographic techniques.^4–6^ Although these methods can provide reliable analytical results, their application require sophisticated instrumentation, relatively extensive sample preparation, lengthy analytical procedures, or skilled operation. Alternatively, electrochemical sensing has consequently emerged as an attractive tool because of its operational simplicity, rapid response, low instrumentation requirements, and compatibility with on-field and portable analytical systems.^7^

Enzyme-based electrochemical sensors have traditionally been used for H_2_O_2_ determination because of the high catalytic efficiency and selectivity of peroxide-reactive enzymes.^8^ However, enzyme immobilization, susceptibility to environmental conditions, storage requirements, and loss of catalytic activity can restrict their operational stability and practical implementation.^9^ These limitations have driven the development of non-enzymatic H_2_O_2_ sensors based on metal nanoparticles, metal oxides, carbon nanomaterials, and their hybrid nanostructures.^10,11^ Thus, the research direction has been shifted from the single-component electrocatalysts toward engineered interfaces that seek to simultaneously improve catalytic activity, electron transport, electroactive surface accessibility, and operational stability.^12–14^

Transition-metal oxides have therefore attracted considerable interest as electrocatalysts for non-enzymatic electrochemical biosensing owing to their redox activity, accessible surface sites, low cost, facile chemical synthesis, and the ability to form nanostructures with high electroactive surface areas.^15^ Among the metal oxides, manganese dioxide (MnO_2_) has received considerable attention for non-enzymatic H_2_O_2_ sensing because of its accessible redox chemistry, chemical stability, low cost, and favorable electrochemical properties.^16,17^ However, the relatively limited electrical conductivity of MnO_2_ can constrain charge transport when it is used as a standalone electrode modifier. This limitation has motivated the development of MnO_2_-based composites with conductive carbon materials and other functional phases.^18^ MnO_2_@carbon nanotube and MnO_2_@graphene-based electrodes have demonstrated the value of coupling the electroactive oxide with conductive carbon frameworks to improve electrical communication within the sensing interface.^19,20^ Therefore, the incorporation of carbon nanomaterials represents an important development in this field. Multi-walled carbon nanotubes (MWCNTs) possess high electrical conductivity, a high aspect ratio, and an interconnected structure that can establish conductive pathways throughout composite electrode layers.^14^ Consequently, MWCNTs-supported metal-oxide architectures have been widely investigated to facilitate electron transport and increase the electrochemical accessibility of active components.^21,22^ However, simply combining an electroactive oxide with a conductive carbon material does not necessarily ensure optimal analytical performance.^23^ Stability of the electrochemical catalytic activity, particle aggregation, limited exposure of active sites, and incomplete electrical contact may still restrict their effective utilization.

Therefore, recent developments have focused on multicomponent and hierarchical architectures in which different materials are introduced to address specific limitations of conventional single- or dual-component electrodes.^24^

In this regard, H_2_O_2_ sensing platforms have incorporated combinations of metal oxides, graphene derivatives, transition-metal compounds, porous materials, and other conductive nanostructures.^25^ This progression reflects a broader shift in the field from maximizing the amount of an electrocatalytic material toward controlling the organization and accessibility of the electrochemical interface. Despite these advances, an important challenge remains in developing composite interfaces that simultaneously provide an electroactive phase, efficient electrical pathways, accessible surface sites, and adequate structural integration while retaining practical applicability to complex food and/or biological matrices. Because increasing the number of components in a sensing material does not, by itself, establish improved performance; hence, the functional components must be effectively integrated and their contribution should be supported by experimental electrochemical evidence. Thus, the development of relatively simple but hierarchically organized composite interfaces, together with systematic electrochemical characterization and real-sample validation, remains relevant to the advancement of non-enzymatic H_2_O_2_ sensing. Within this context, selenium dioxide (SeO_2_) provides an additional inorganic component with distinct physicochemical and electrochemical characteristics.^26^

Accordingly, the present study develops a hierarchical MCC/MnO_2_–SeO_2_@MWCNTs nanocomposite as a non-enzymatic electrochemical interface for H_2_O_2_ determination using a carbon paste electrode. The proposed architecture integrates an MnO_2_–SeO_2_ mixed-oxide phase with an interconnected MWCNTs network within an MCC-supported matrix. The nanocomposite and its individual components were systematically characterized by Fourier-transform infrared spectroscopy (FTIR), transmission electron microscopy (TEM), cyclic voltammetry (CV), electrochemical impedance spectroscopy (EIS), and chronoamperometry (CA). The resulting electrode was evaluated with respect to its electrochemically active surface area, amperometric response, linear range, detection limit, selectivity, repeatability, reproducibility, and storage stability. Accordingly, analytical performance and practical applicability of the proposed hierarchical composite interface was successfully tested for non-enzymatic H_2_O_2_ determination in diverse food matrices.

## 2. Materials and methods

### 2.1 Chemicals and reagents

Multi-walled carbon nanotubes (MWCNTs, >95% purity, Sigma-Aldrich), solution of H_2_O_2_ (50%, Za-Chem), phosphate-buffered saline (PBS, Tablets, LOBA Chemie), were used. Potassium ferrocyanide, potassium ferricyanide, hydrochloric acid (HCl), sodium hydroxide (NaOH), and hydrogen peroxide were purchased from PioChem, Egypt. All other chemicals were of analytical grade, and all solutions were prepared using doubly distilled water without any further purification.

### 2.2 Preparation of microcrystalline cellulose (MCC)

The MCC powder was prepared from cellulose extracted from citrus waste by acid hydrolysis. Briefly, the extracted cellulose was treated with 2.0 M sulfuric acid under reflux for 45 min at a solid-to-liquid ratio of 1 : 10 (w/v). The resulting acid-hydrolyzed cellulose was recovered and thoroughly washed with double-distilled water until the residual acidity was removed, followed by washing with acetone. The washed material was then dried until a constant solid weight was obtained. The resulting MCC powder was collected and stored for subsequent physicochemical characterization and preparation of the nanocomposite. The MCC yield was approximately 30%, calculated based on the initial mass of citrus waste.

### 2.3 Preparation of MCC/MnO_2_–SeO_2_@MWCNTs nanocomposite

The MCC/MnO_2_–SeO_2_@MWCNTs nanocomposite was prepared using a one-pot ultrasonic-assisted assembly method followed by low-temperature drying. Briefly, MCC (5.0 mg) was dispersed in 1.0 mL of double-distilled water using a water-bath sonicator for 15 min at room temperature. Subsequently, equal volumes of SeO_2_, MnO_2_, and MWCNTs dispersions (5.0 mg mL^−1^ each) were added to the MCC suspension under continuous magnetic stirring at 600 rpm. The mixture was then stirred for an additional 5.0 min to promote homogeneous dispersion and physical association of the oxides and carbon nanotubes within the MCC matrix. The resulting suspension was dried to obtain the MCC/MnO_2_–SeO_2_@MWCNTs composite powder, which was stored for subsequent characterization and electrode modification.

### 2.4 Morphological and chemical characterization

Morphology of the prepared materials was examined using field-emission scanning electron microscopy (FE-SEM, Hitachi S-4800) operated at an accelerating voltage of 5.0–10 kV. Transmission electron microscopy (TEM, JEOL JEM-2100) operated at 200 kV was employed to investigate the nanoscale morphology, particle distribution, and dispersion of the components within the nanocomposite. Chemical identification were analyzed with the Fourier-transform infrared (FTIR), whereas all spectra were recorded over the range of 4000–400 cm^−1^ using a Thermo Scientific Nicolet iS10.

### 2.5 Carbon paste preparation and electrode surface modification

A carbon paste electrode (CPE) was fabricated and subsequently modified with the prepared MCC/MnO_2_–SeO_2_@MWCNTs nanocomposite for electrochemical evaluation. The CPE was firstly prepared by thoroughly mixing graphite powder (1.0 g) with paraffin oil (250 µL) to obtain a homogeneous carbon paste. The paste was packed into the electrode body, and a copper wire was inserted to provide electrical contact. The exposed electrode surface was smoothed by polishing on weighing filter paper to obtain a uniform and reproducible surface.

For surface modification, 5.0 mg of each of MCC, MnO_2_, SeO_2_, or MCC/MnO_2_–SeO_2_@MWCNTs nanocomposite was separately dispersed in 1.0 mL of double-distilled water and sonicated for 30 min. Subsequently, 30 µL of each suspension was drop-cast onto the CPE surface and allowed to dry at room temperature for 1–2 h. The corresponding electrodes were designated CPE/MCC, CPE/MnO_2_, CPE/SeO_2_, and CPE/MCC/MnO_2_–SeO_2_@MWCNTs, respectively. Prior electrochemical measurements, modified electrodes were rinsed with PBS to remove unattached materials and then used for electrochemical measurements. Before electrochemical measurements, the working electrodes were activated by five consecutive voltammetric cycles in PBS (pH 7.0) over the potential range of −0.7 to +1.0 V *versus* Ag/AgCl. All electrochemical measurements were performed at room temperature.

### 2.6 Electrochemical measurements

Electrochemical measurements were performed using a PalmSens potentiostat (PalmSens-4 handled device) in a conventional three-electrode configuration consisting of the MCC/MnO_2_–SeO_2_@MWCNTs/CPE as the working electrode, an Ag/AgCl reference electrode, and a platinum-wire counter electrode. Cyclic voltammetry (CV) was performed over the potential range of −0.7 to +1.0 V at a scan rate of 50 mV s^−1^. CV and electrochemical impedance spectroscopy (EIS) were used to characterize the interfacial electron-transfer characteristics, and the electrochemical behavior of the modified electrodes using a 5.0 mM [Fe(CN)_6_]^3−^/^4−^ redox probe. All measurements were conducted at room temperature (25 ± 2.0 °C).

Chronoamperometric detection of H_2_O_2_ was performed at a constant potential of +0.7 V *versus* Ag/AgCl in phosphate-buffered saline (PBS, pH 7.0), with successive additions of H_2_O_2_. The resulting calibration curve was used to determine the analytical sensitivity, linear response range, LOD, LOQ, and response time. The limit of detection (LOD), and limit of quantification (LOQ) were calculated as LOD = 3*σ*/*S* and LOQ = 10*σ*/*S*, respectively, where *σ* is the standard deviation of the blank response (*N* = 5) and *S* is the slope of the calibration curve. The response time was determined from the chronoamperometric current–time response following H_2_O_2_ addition.

### 2.7 Real-sample preparation and analysis

Food samples, including yellow cheese, salami, beef, and milk, were collected from local markets in Egypt. For solid food samples, approximately 5.0 g of each homogenized sample was dried and grinded, followed by extraction with 50 mL of phosphate buffer (pH 7.0). The mixtures were stirred and sonicated in a thermostatically controlled ultrasonic bath at 75 °C for 25 min. The extracts were then filtered through Whatman filter paper to remove suspended solid residues, and the filtrates were collected for subsequent electrochemical analysis.

For milk samples, the samples were filtered directly through Whatman filter paper. For electrochemical analysis, 500 µL of each filtered extract or milk sample was diluted with 4500 µL of phosphate buffer (pH 7.0), and the resulting solution was analyzed by chronoamperometry at +0.7 V *versus* Ag/AgCl.

For recovery studies, known amounts of H_2_O_2_ were added to the samples, and the recovery was calculated as:Recovery (%) = [(*C*_spiked_ − *C*_unspiked_)/*C*_added_] × 100where *C*_spiked_ is the H_2_O_2_ concentration measured in the spiked sample, *C*_unspiked_ is the concentration measured in the non-spiked sample, and *C*_added_ is the standard added concentration of H_2_O_2_ added. Results were expressed as mean ± standard deviation (SD).

## 3. Results and discussion

### 3.1 Material characterization

To define the most important acquired physical and chemical features by the implemented composite, several characterization techniques were conducted. In this regard, the FTIR spectrum of MCC exhibited a broad absorption band at approximately 3358 cm^−1^, assigned to the O–H stretching vibrations, while the band near 2855 cm^−1^ was attributed to C–H stretching vibrations of the cellulose backbone. The absorption band around 1620 cm^−1^ was associated with the bending vibration of adsorbed water. Characteristic cellulose bands were observed at approximately 1430 cm^−1^ (CH_2_ scissoring), 1370 cm^−1^ (C–H bending), 1160 cm^−1^ (asymmetric C–O–C stretching), 1055 cm^−1^ (C–O stretching), and 898 cm^−1^ (β-glycosidic linkage-associated vibration), consistent with the characteristic FTIR profile of cellulose.

Furthermore, the FTIR spectrum of MnO_2_ exhibited a broad band around 3410 cm^−1^ and a weaker band near 1620 cm^−1^, corresponding to O–H stretching and H–O–H bending vibrations, respectively, mainly associated with adsorbed water molecules. Several absorption bands in the low-wavenumber region, particularly those around 579, 630, and 709 cm^−1^, were attributed to Mn–O and Mn–O–Mn vibrations, supporting the presence of manganese oxide.

Additionally, the FTIR spectrum of SeO_2_ exhibited characteristic absorption bands in the approximately 850–890 cm^−1^ region, attributable to Se–O stretching vibrations. Other FTIR bands in the region of 720–780 cm^−1^ associated with Se–O vibrations of the selenium dioxide structure were collected. Weak bands near 3400 and 1630 cm^−1^ were attributed to O–H stretching and H–O–H bending vibrations, respectively, associated with adsorbed moisture.

MWCNTs exhibited a broad absorption band at approximately 3440 cm^−1^, corresponding to O–H stretching vibrations associated with surface oxygen-containing groups and/or adsorbed moisture. The bands near 2920 and 2850 cm^−1^ were attributed to aliphatic C–H stretching vibrations. The absorption feature around 1630 cm^−1^ may be associated with graphitic C=C vibrations and/or adsorbed water, whereas bands in the 1000–1800 cm^−1^ region may arise from oxygen-containing surface functionalities. The bands near 1440, 1380, and 1110 cm^−1^ were assigned to carbon-framework and C–O-related vibrations, respectively.

Finally, the FTIR spectrum of the MCC/MnO_2_–SeO_2_@MWCNTs nanocomposite retained characteristic absorption features of the individual constituents, including the O–H stretching region, cellulose-related vibrations, Mn–O vibrations, Se–O vibrations, and characteristic MWCNTs-related absorptions. The broad O–H band appeared in the approximately 3330–3400 cm^−1^ region and exhibited changes in position and bandwidth compared with MCC, indicating a modified hydrogen-bonding environment following incorporation of the inorganic and carbon components. The cellulose-related bands around 1148, 1010, and 834 cm^−1^ remained detectable, although their relative intensities changed. Absorptions in the 500–700 cm^−1^ region were consistent with all Mn–O vibrations, whereas bands in the approximately 750–890 cm^−1^ region were correlated with the Se–O vibrations. The characteristic carbon-related absorption features of the MWCNTs were also retained in the composite spectrum.

The observed changes in peak positions and relative band intensities indicate changes in the local chemical environment of the constituent materials and are consistent with their incorporation into the MCC/MnO_2_–SeO_2_@MWCNTs composite. Hence, these FTIR results (Fig. 1A) supported the presence and integration of the principal chemical components within the prepared composite.

**Fig. 1:**
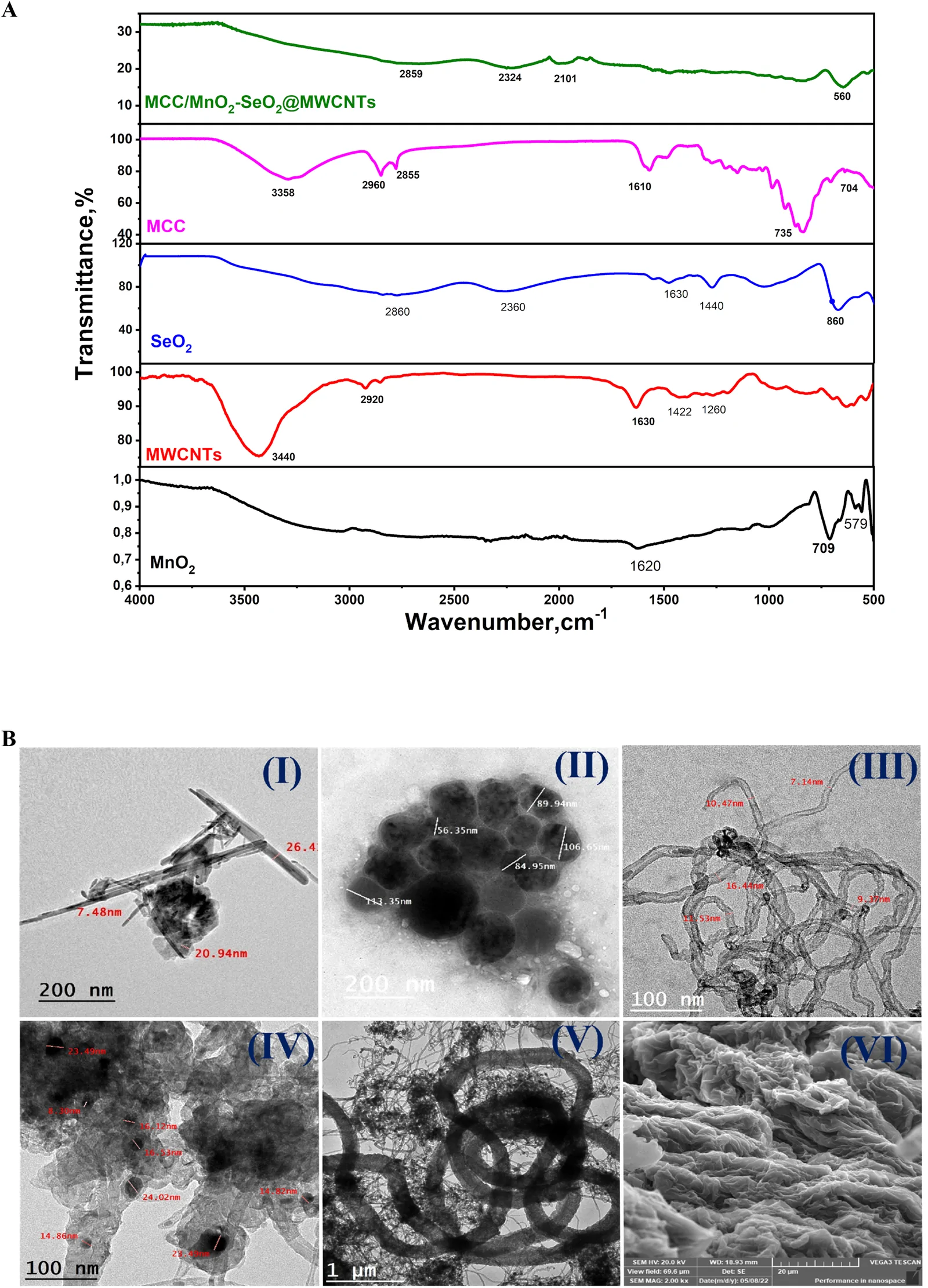
(A) FTIR Spectra of MCC, MnO_2_, SeO_2_, MWCNTs and the MCC/MnO_2_–SeO_2_@MWCNTs nanocomposite. (B) TEM&SEM images for the prepared materials: (I) MnO_2_, (II) SeO_2_, (III) MWCNTs, (IV) MnO_2_–SeO_2_, (V) MnO_2_–SeO_2_@MWCNTs, and (VI) MCC (SEM image).

The morphological characteristics and microstructural features of the prepared materials were further investigated (Fig. 1B) using transmission electron microscopy (TEM) and scanning electron microscopy (SEM). The TEM image of MnO_2_ revealed predominantly rod-like nanoscale structures with some degree of aggregation, consistent with the morphology reported for MnO_2_ prepared by related precipitation methods. SeO_2_ exhibited nanoscale particulate features with relatively uniform dispersion in the examined region. The TEM image of MWCNTs showed the characteristic hollow tubular morphology with a high aspect ratio, consistent with the expected structure of multi-walled carbon nanotubes.

The SeO_2_/MWCNTs composite showed SeO_2_ nanoparticles associated with the surfaces of the nanotubes, although some degree of particle aggregation was still evident. The MnO_2_–SeO_2_ binary material exhibited close spatial association between the MnO_2_ and SeO_2_ nanostructures, producing a heterogeneous composite morphology. Following incorporation of MWCNTs, the MnO_2_–SeO_2_@MWCNTs material displayed an interconnected nanotube-based morphology, with MnO_2_ and SeO_2_ particulate domains distributed along the carbon framework. These observations are consistent with the formation of a multicomponent composite containing a continuous carbon-nanotube network.

TEM examination of the MCC/MnO_2_–SeO_2_@MWCNTs nanocomposite further demonstrated the coexistence of MCC-associated structures, MWCNTs, and nanoscale MnO_2_ and SeO_2_ domains within the examined regions. The inorganic nanoparticles appeared to be distributed throughout the carbon-containing network and associated with the MCC matrix, indicating successful physical integration of the composite components. The MCC therefore serves as a supporting matrix within the multicomponent structure.

SEM examination of MCC revealed an irregular fibrous morphology with rough surface features characteristic of cellulose-derived materials. The observed morphology provides a suitable structural matrix for incorporation of the bimetal oxides and carbon nanotubes components. Therefore, the FTIR, TEM, and SEM results support the incorporation and morphological association of MCC, MnO_2_, SeO_2_, and MWCNTs within the prepared composite. The resulting multicomponent architecture provides a suitable structural basis for subsequent electrochemical evaluation.

### 3.2 Electrochemical characterization

The electrochemical behavior of the bare and modified CPEs was investigated by the CV and the EIS using the [Fe(CN)_6_]^3−/4−^ as a redox probe. As shown in Fig. 2A, the bare electrode exhibited a relatively weak and less reversible redox response, indicative of limited heterogeneous electron-transfer kinetics at the electrode/electrolyte interface. Modification with MCC enhanced the redox-current response and altered the peak separation, suggesting improved accessibility of the electrode interface to the redox probe. This improvement is likely associated with changes in surface morphology, wettability, and interfacial organization introduced by the cellulose matrix rather than with an increase in intrinsic electrical conductivity.

**Fig. 2:**
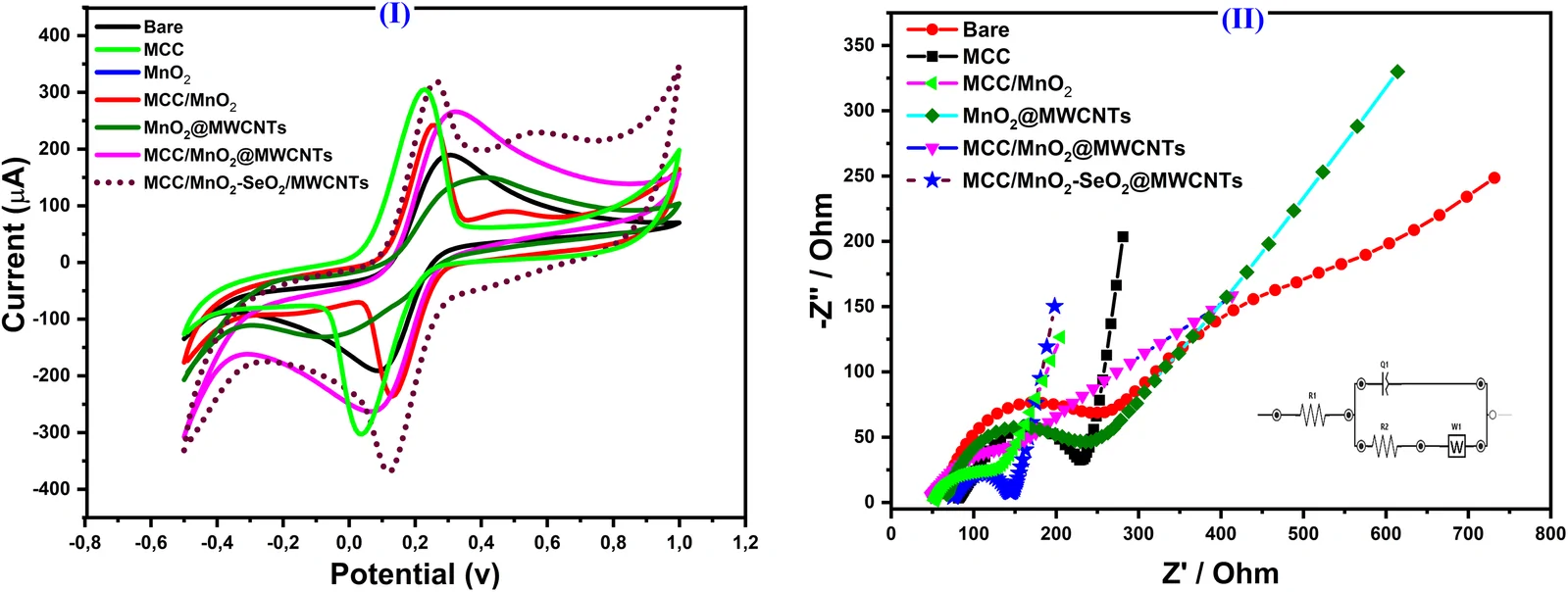
(B) (I) Cyclic voltammetry and (II) electrochemical impedance spectroscopic analysis of the different CPE-modified electrodes in a solution containing ferro/ferricyanide and PBS. The CV measurements were performed at a scan rate of 50 mV s^−1^. The inset in (II) demonstrates the equivalent electrical Randles-circuit used for fitting the EIS data.

Incorporation of MnO_2_ led to a further enhancement to the voltammetric response, reflecting the contribution of the electroactive manganese metal-oxide phase to the interfacial electrochemical behavior. The MnO_2_@MWCNTs electrode did not exhibit a proportional enhancement in the redox response, indicating that the incorporation of conductive nanotubes alone does not necessarily ensure optimal electrochemical performance. In contrast, the combined MCC/MnO_2_@MWCNTs architecture produced a stronger redox response, highlighting the importance of the spatial integration of the supporting matrix, metal oxide, and conductive carbon phase. The MCC/MnO_2_–SeO_2_@MWCNTs-modified electrode exhibited the most pronounced redox response among the investigated electrodes, indicating a highly accessible and electrochemically active interface. Its favorable voltammetric behavior can be attributed to the complementary functions of the composite components. The MnO_2_–SeO_2_ metal-oxide phase provides electroactive sites, the MWCNTs network facilitates electronic conduction, and the MCC matrix promotes the structural integration and dispersion of the inorganic and carbon components. The resulting architecture therefore facilitates interaction between the redox probe and the electrode surface. In parallel to the CV, EIS measurements were subsequently performed to further evaluate the interfacial charge-transfer characteristics of the electrodes. The Nyquist plots in Fig. 2B showed impedimetric features associated with the uncompensated solution resistance, interfacial charge-transfer process, and diffusion-related impedance. The spectra were fitted using the equivalent electrical circuit shown as an inset in Fig. 2B, and the corresponding electrochemical parameters derived from the CV and EIS measurements are summarized in Table S1 (SI), providing a quantitative comparison of the electrochemical behavior of the investigated electrodes. The bare electrode exhibited the greatest charge-transfer resistance, consistent with relatively slow heterogeneous electron transfer. The MCC modification substantially decreased the charge-transfer resistance, supporting the improved interfacial accessibility observed by the CV.

Notably, the MCC/MnO_2_–SeO_2_@MWCNTs-modified electrode displayed the lowest charge-transfer resistance among all investigated electrodes, indicating the most favourable heterogeneous charge-transfer characteristics. This behavior is attributed to the incorporation of the MnO_2_–SeO_2_ metal-oxide phase within the conductive MWCNTs network, supported by the MCC matrix, effectively reducing interfacial electron-transfer limitations.

The MCC primarily functions as a renewable supporting matrix, providing a structural framework for the integration and distribution of the active components. MWCNTs establish conductive pathways that facilitate electron transport between the electroactive domains and the underlying electrode. MnO_2_ provides electroactive metal-oxide sites, while SeO_2_ introduces an additional metal-oxide component that can contribute to the interfacial electrochemical environment and the overall electroactive architecture. Their integration within a single composite is therefore expected to improve the connectivity between electroactive domains and the electrode surface.

Thus, the CV and EIS analysis confirmed that the MCC/MnO_2_–SeO_2_@MWCNTs composite provides a more favorable electrochemical interface than the bare and partially modified electrodes. The combination of a structurally supportive MCC matrix, an electroactive MnO_2_–SeO_2_ metal-oxide phase, and an electrically conductive MWCNTs network provides a rational basis for enhanced interfacial charge transfer and electrochemical accessibility. These characteristics support the suitability of the composite as an electrode modifier for subsequent non-enzymatic electrochemical determination of H_2_O_2_.

### 3.3 Determination of electrochemically active surface area

The electrochemically active surface area (ECSA) of the unmodified and modified electrode surfaces with the MCC/MnO_2_–SeO_2_@MWCNTs was evaluated using the scan rate effects conducted by CV measurements in the utilized redox probe ([Fe(CN)_6_]^3−/4−^, 5.0 mM), as shown in Fig. 3A & C. For both electrodes, the anodic and cathodic peak current increased linearly with the square root of the scan rate (Fig. 3B & D), indicating that the electrochemical response was predominantly governed by the diffusion of the redox probe to the electrode surface. Accordingly, the ECSA was calculated from the linear relationship giving by the Randles–Ševčík equation:*I*_p_ = (2.69 × 10^5^)*n*^3/2^*AD*^1/2^*Cν*^1/2^where *I*_p_ is the peak current (A), *n* is the number of electrons transferred (one-electron for the ([Fe(CN)_6_]^3−/4−^), *A* is the targeting electrochemically active surface area (cm^2^), *D* is the diffusion coefficient of the redox probe (7.6 × 10^−6^ cm^2^ s^−1^), *C* is the concentration of the redox probe (5 × 10^−6^ mol cm^−3^), and *ν* is the scan rate (V s^−1^).

**Fig. 3:**
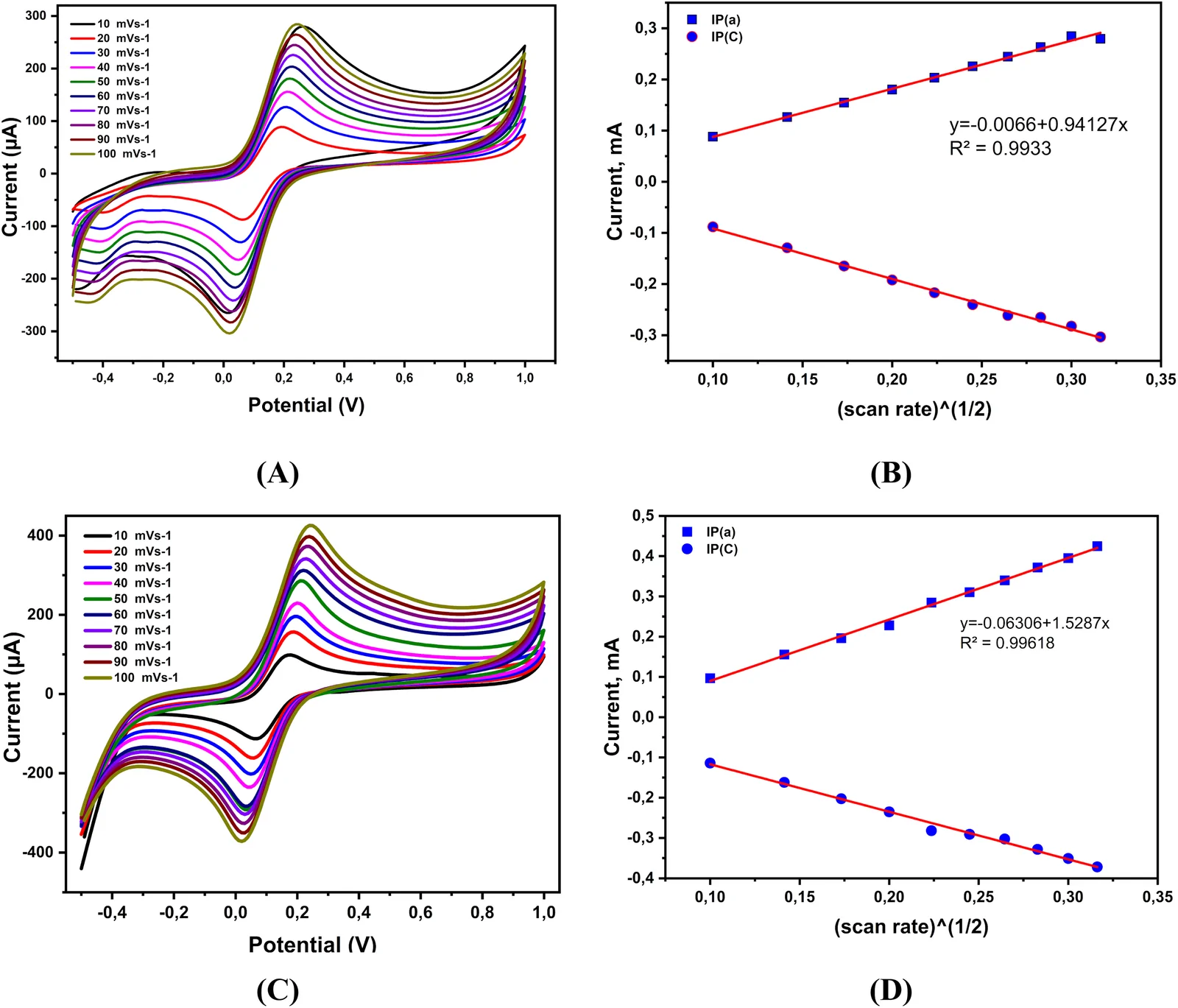
Cyclic voltammograms recorded at different scan rates for (A) the bare electrode and (C) the MCC/MnO_2_–SeO_2_@MWCNTs-modified electrode, with the corresponding linear plots of anodic and cathodic peak currents (*I*_pa_, and *I*_pc_) *versus* the square root of the scan rate for (B) the bare electrode and (D) the MCC/MnO_2_–SeO_2_@MWCNTs-modified electrode. The CV Measurements were performed using 5.0 mM [Fe(CN)_6_]^3−/4−^ in 0.1 M PBS as the supporting electrolyte.

Thus, the MCC/MnO_2_–SeO_2_@MWCNTs-based electrode exhibited an ECSA of 0.412 cm^2^, substantially higher than that of the bare electrode (0.253 cm^2^), corresponding to an approximately 63% increase in electrochemically active surface area. This surface expansion is contributed to the synergistic enhancement of electrochemical reactivity that resulted from the electrically conductive MWCNTs network, nanostructured and electrocatalytic reactivity of MnO_2_ and SeO_2_, or the porous MCC matrix. The porous MCC framework provides an interconnected structure that facilitates electrolyte penetration and access of the redox probe to the electrode interface, while the dispersed MnO_2_ and SeO_2_ nanostructures increase the number of accessible interfacial sites. Meanwhile, the MWCNTs network enables the conductive pathways that allows electron transport and promotes effective dispersion of the inorganic nanostructures within the composite. From the perspective of peroxide electrochemical analysis, this remarkable increase in the ECSA will provide a greater number of sites for electrochemical reactions involving H_2_O_2_ and contribute to the enhanced current response and analytical sensitivity of the developed sensor. Together with the complementary electrochemical and physicochemical characterization, these findings support the successful development of the nanostructured electrode interface for H_2_O_2_ sensing.

### 3.4 Electrochemical detection of hydrogen peroxide

The electrochemical responses of the fabricated MCC/MnO_2_–SeO_2_@MWCNTs-modified CPEs toward H_2_O_2_ were initially evaluated. To assess the effect of the individual components and their integration, electrodes modified with MnO_2_, SeO_2_, MnO_2_–SeO_2_, MnO_2_–SeO_2_/MWCNTs, and MCC/MnO_2_–SeO_2_@MWCNTs were investigated (Fig. 4A). Each electrode was exposed to the investigated H_2_O_2_ concentrations, and the resulting oxidation currents were recorded and comparatively evaluated.

**Fig. 4:**
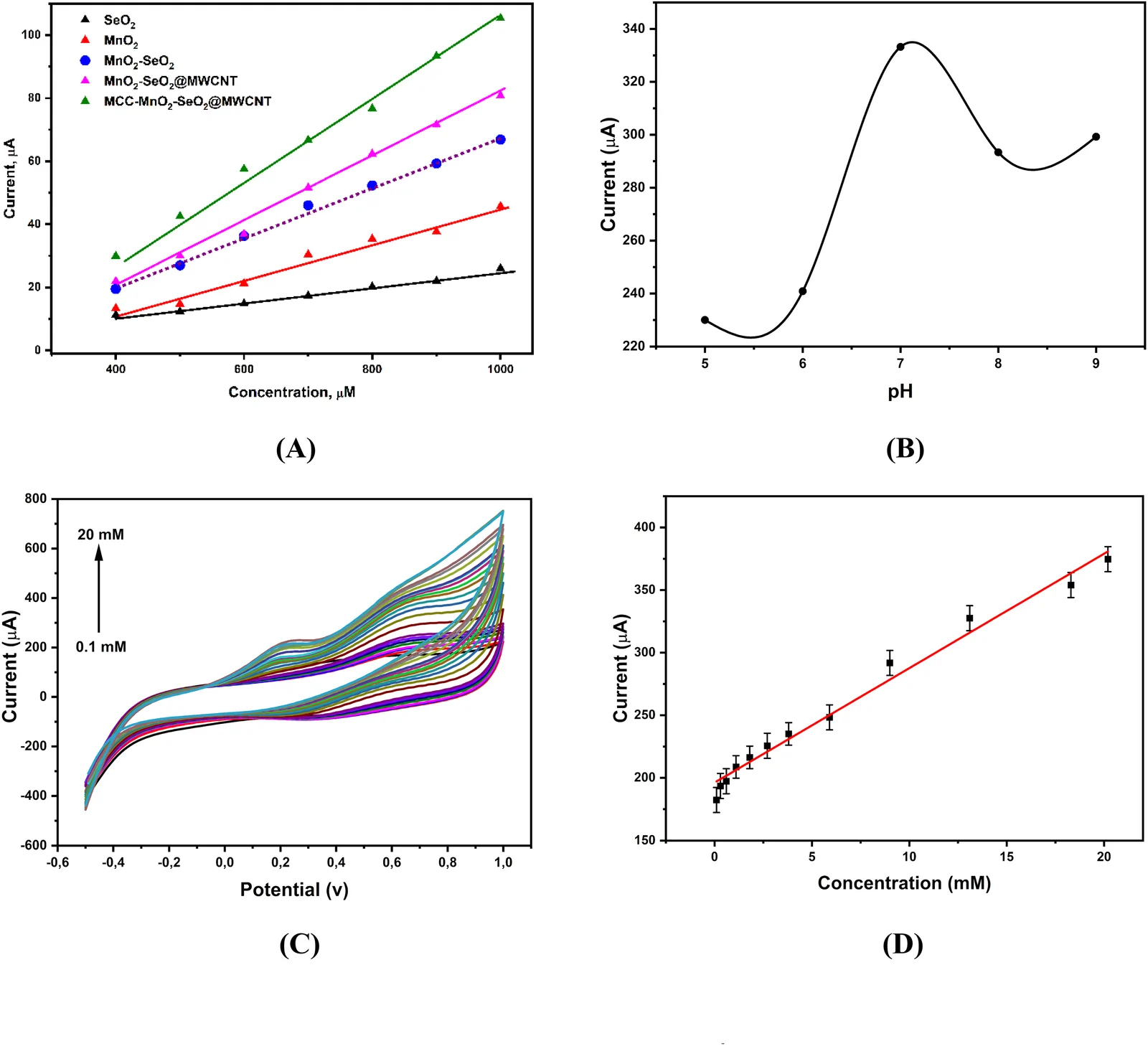
(A) Chronoamperometric (CA) responses of MnO_2_, SeO_2_, MnO_2_–SeO_2_, MnO_2_–SeO_2_/MWCNTs, and MCC/MnO_2_–SeO_2_@MWCNTs-modified electrodes toward different concentrations of H_2_O_2_. (B) Effect of PBS pH on the oxidation current response of the MCC/MnO_2_–SeO_2_@MWCNTs-modified electrode toward H_2_O_2_. (C) Cyclic voltammograms of the MCC/MnO_2_–SeO_2_@MWCNTs-modified electrode recorded in PBS (pH 7.0) with increasing H_2_O_2_ concentrations from 0.1 to 20 mM. (D) Corresponding linear calibration plot of the oxidation current *versus* H_2_O_2_ concentration.

The MnO_2_- and SeO_2_-modified electrodes exhibited lower oxidation-current responses than the MnO_2_–SeO_2_-modified electrode, indicating a higher H_2_O_2_ response for the combined oxide phase under the conditions investigated. Incorporation of MWCNTs into the MnO_2_–SeO_2_ system resulted in a further increase in the measured current response, while the MCC/MnO_2_–SeO_2_@MWCNTs-modified electrode exhibited the highest response among the evaluated configurations. These comparative measurements provide direct experimental evidence that the complete MCC/MnO_2_–SeO_2_@MWCNTs configuration produces a greater electrochemical response toward H_2_O_2_ than the individual components and the corresponding partially integrated configurations under the same experimental conditions.

The pH of the supporting electrolyte is an important parameter in H_2_O_2_ electrochemical sensing because it can influence the interfacial charge, proton-coupled electrochemical processes, and surface chemistry of the modified electrode. Accordingly, the response of the MCC/MnO_2_–SeO_2_@MWCNTs-modified electrode was evaluated over a pH range of 5.0–9.0 using PBS as the supporting electrolyte. As shown in Fig. 4B, the oxidation current reached its maximum at pH 7.0, which was therefore selected as the optimum condition for subsequent measurements. The lower current response observed at pH 5.0–6.0 might be associated with pH-dependent changes in the protonation state of surface functional groups and the resulting interfacial environment, which can affect the accessibility and reactivity of electrochemically active sites. The decrease in current at pH 8.0–9.0 may likewise be related to changes in the surface charge and interfacial reaction environment. Therefore, pH 7.0 was selected for subsequent H_2_O_2_ measurements.

Following pH optimization, the electrochemical response of the modified electrode toward different H_2_O_2_ concentrations was evaluated by CV in PBS (pH 7.0) over the potential range of −0.5 to +1.0 V. In the absence of H_2_O_2_, the modified electrode exhibited a relatively low background current without a pronounced faradaic response within the investigated potential window. Upon successive addition of H_2_O_2_, the anodic current increased progressively, as shown in Fig. 4C, demonstrating a concentration-dependent electrochemical response toward H_2_O_2_. The corresponding calibration relationship between the oxidation current and H_2_O_2_ concentration is presented in Fig. 4D. A linear response was obtained over the concentration range of 0.1–20 mM, with a reported limit of detection of 0.05 mM. These results demonstrate the applicability of the modified electrode for quantitative H_2_O_2_ determination within the investigated concentration range.

The enhanced electrochemical response can be attributed to the complementary contributions of the components of the MCC/MnO_2_–SeO_2_@MWCNTs composite. The MnO_2_ and SeO_2_ nanostructured components contribute to the electrochemically active inorganic interface, while the MWCNTs network provides conductive pathways that facilitate charge transport across the modified electrode. The MCC matrix provides a porous supporting framework that promotes dispersion of the nanostructured components and facilitates electrolyte and H_2_O_2_ access to the electrode/electrolyte interface. Furthermore, the larger electrochemically active surface area determined for the composite-modified electrode provides a greater accessible interfacial area, which can contribute to the increased electrochemical current response. The observed concentration-dependent increase in current and the resulting linear calibration relationship support the applicability of the MCC/MnO_2_–SeO_2_@MWCNTs-modified electrode as a non-enzymatic electrochemical platform for H_2_O_2_ detection.

### 3.5 Chronoamperometric measurements

The amount of nanocomposite deposited on the electrode surface is an important parameter that can influence the electrochemical and analytical performance of the sensor by affecting the accessible electrochemical surface area, mass transport, interfacial charge transfer, and accessibility of electroactive sites. In this study, 30 µL of aqueous MCC/MnO_2_–SeO_2_@MWCNTs nanocomposite dispersions at concentrations of 2, 3, 5, 10, 15, 20, and 25 mg mL^−1^ were investigated to determine the optimum loading on the carbon paste electrode. Among the tested concentrations, the electrode modified with 30 µL of the 5.0 mg mL^−1^ nanocomposite dispersion exhibited the highest and most stable current response toward H_2_O_2_, indicating a favorable balance between the amount of electrochemically accessible material and mass transport at the electrode interface (Fig. 5A). The lower nanocomposite concentrations produced lower current responses, which may be associated with insufficient surface coverage and a lower density of accessible electroactive sites. In contrast, increasing the nanocomposite concentration to 10–25 mg mL^−1^ resulted in a decrease in the current response. This behavior may be attributed to the formation of a thicker or more compact surface layer at higher loadings, which can hinder mass transport and increase the resistance of the modified interface. Similar effects have been reported for MnO_2_-based composite electrodes, where excessive material loading can adversely affect H_2_O_2_ sensing performance. Therefore, 30 µL of the 5.0 mg mL^−1^ nanocomposite dispersion was selected as the optimum loading condition for subsequent measurements.

**Fig. 5:**
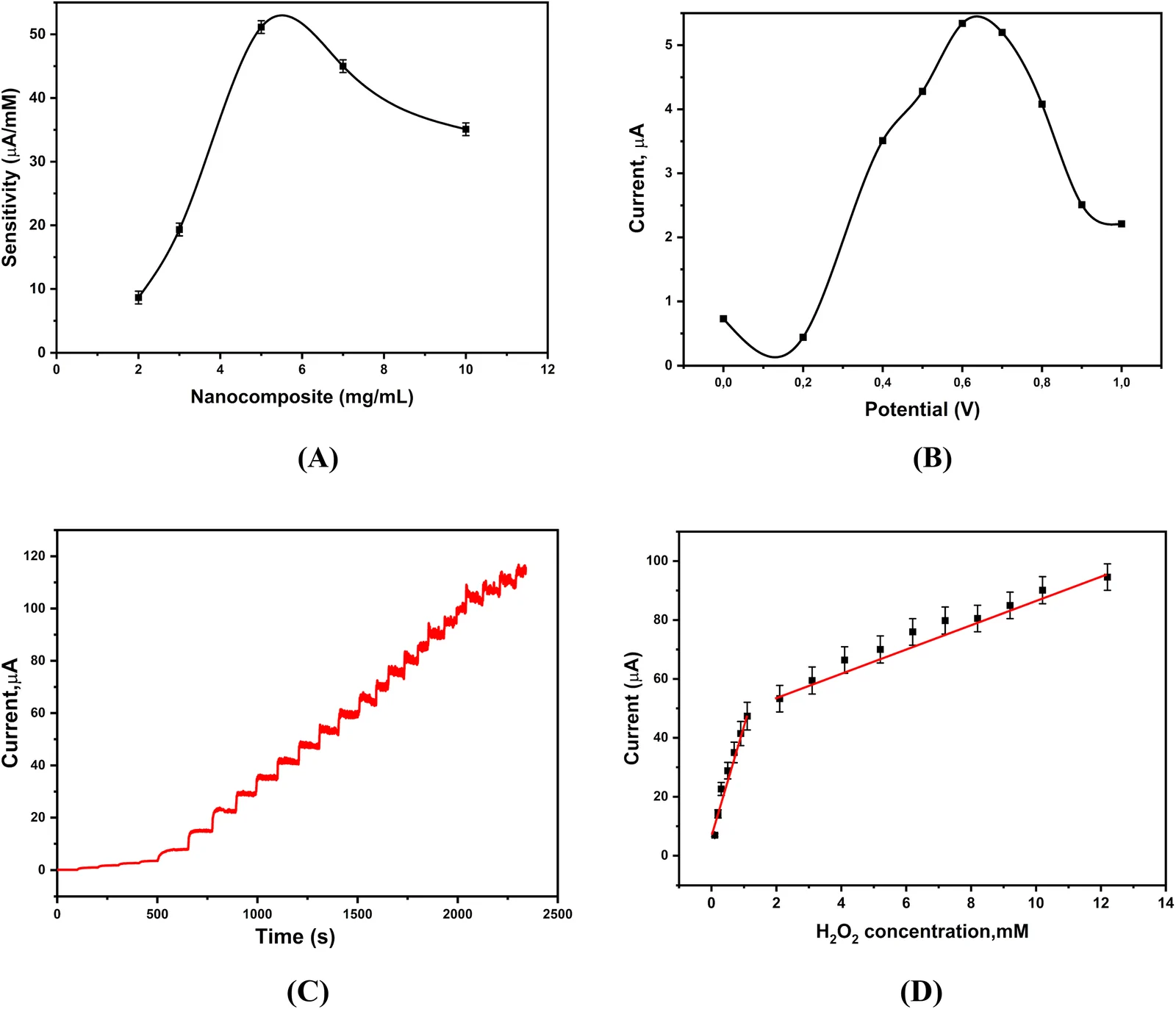
(A) Effect of MCC/MnO_2_–SeO_2_@MWCNTs nanocomposite concentration on the current response of the modified electrode toward H_2_O_2_. (B) Effect of the applied potential on the current response of the MCC/MnO_2_–SeO_2_@MWCNTs-modified electrode toward 0.1 µM H_2_O_2_ in PBS (pH 7.0). (C) Chronoamperometric response of the MCC/MnO_2_–SeO_2_@MWCNTs-modified electrode to successive additions of H_2_O_2_ in PBS (pH 7.0) at an applied potential of +0.7 V. (D) Corresponding linear calibration plot of the current response *versus* H_2_O_2_ concentration.

The chronoamperometric response of the developed sensor toward H_2_O_2_ was subsequently evaluated under the optimized experimental conditions. The current response was continuously monitored following successive additions of H_2_O_2_ to PBS (pH 7.0), and the resulting current–time profiles were recorded (Fig. 5B and C). Each successive addition of H_2_O_2_ produced a distinct increase in the current response, demonstrating a concentration-dependent electrochemical response of the modified electrode. The rapid establishment of a measurable current response following the successive additions indicates favorable response characteristics under the applied experimental conditions. The current response increased progressively with increasing H_2_O_2_ concentration, and the corresponding calibration relationship exhibited linear behavior over the investigated concentration ranges. The MCC/MnO_2_–SeO_2_@MWCNTs-modified electrode showed reported linear response ranges of 10–1000 µM (sensitivity = 38.25 µA mM^−1^) and 2000–1200 µM ((sensitivity = 4.025 µA mM^−1^), together with a limit of detection of 1.0 µM at a signal-to-noise ratio (S/N) of 3. These results demonstrate the applicability of the developed modified electrode for quantitative H_2_O_2_ determination over the investigated concentration range.

Additionally, Table 1 has been added to demonstrate the performance of the newly developed electrochemical approach for hydrogen peroxide detection in comparison with selected previously reported electrochemical methods based on nanostructured electrode modifiers.

**Table 1:** Comparison of the proposed MCC/MnO_2_–SeO_2_/MWCNTs-based CPE sensor with some previously reported electrochemical peroxide detections relying on nanostructured materials

Modified material	Applied potential (V)	Sensitivity	Linear range (µM)	Detection limit (µM)	Sample matrix	Ref.
Co_3_O_4_-NWs/CFa	—	230 nA µM^−1^ cm^−2^	10–1400	1.4	—	29
Cu_2_O/graphene	0.7	0.285 mA mM^−1^ cm^−2^	30–7800	20.8	—	30
Pt–ZnO nanotube	−0.7	−3.9402 µA mM^−1^	20–5000	1.5	—	31
NiCo_2_S_4_/rGO	−0.45	118.5 µA mM^−1^ cm^−2^	25–11 250	0.19	Milk and toner	32
Pt/Fe_3_O_4_/rGO	0.0	6.875 µA mM^−1^	100–2400	1.58	—	33
CoFe_2_O_4_	−0.45 V	17 nA µM^−1^ cm^−2^	10–1200	2.5	Human urine	34
NiO/α-Fe_2_O_3_	0.4 V	146.98 µA µM^−1^ cm^−2^	500–3000	50	—	35
MCC/MnO_2_–SeO_2_@MWCNTs	0.6	38.25 µA mM^−1^	10–1200	1	Milk & food	This work

### 3.6 Effect of scan rate and evaluation of electron-transfer kinetics

The effect of scan rate on the electrochemical oxidation of H_2_O_2_ at the modified CPE was investigated to assess the mass-transport characteristics and electron-transfer kinetics of the electrode process. Cyclic voltammograms were recorded at scan rates ranging from 50 to 1000 mV s^−1^ under the optimized experimental conditions and at a fixed H_2_O_2_ concentration. As shown in Fig. 6A, the anodic peak current (*I*_pa_) increased progressively with increasing scan rate. A linear relationship was obtained between *I*_pa_ and *v*^1/2^ (Fig. 6B), indicating that diffusion of H_2_O_2_ from the bulk solution to the electrode interface makes a substantial contribution to the measured current response.

**Fig. 6:**
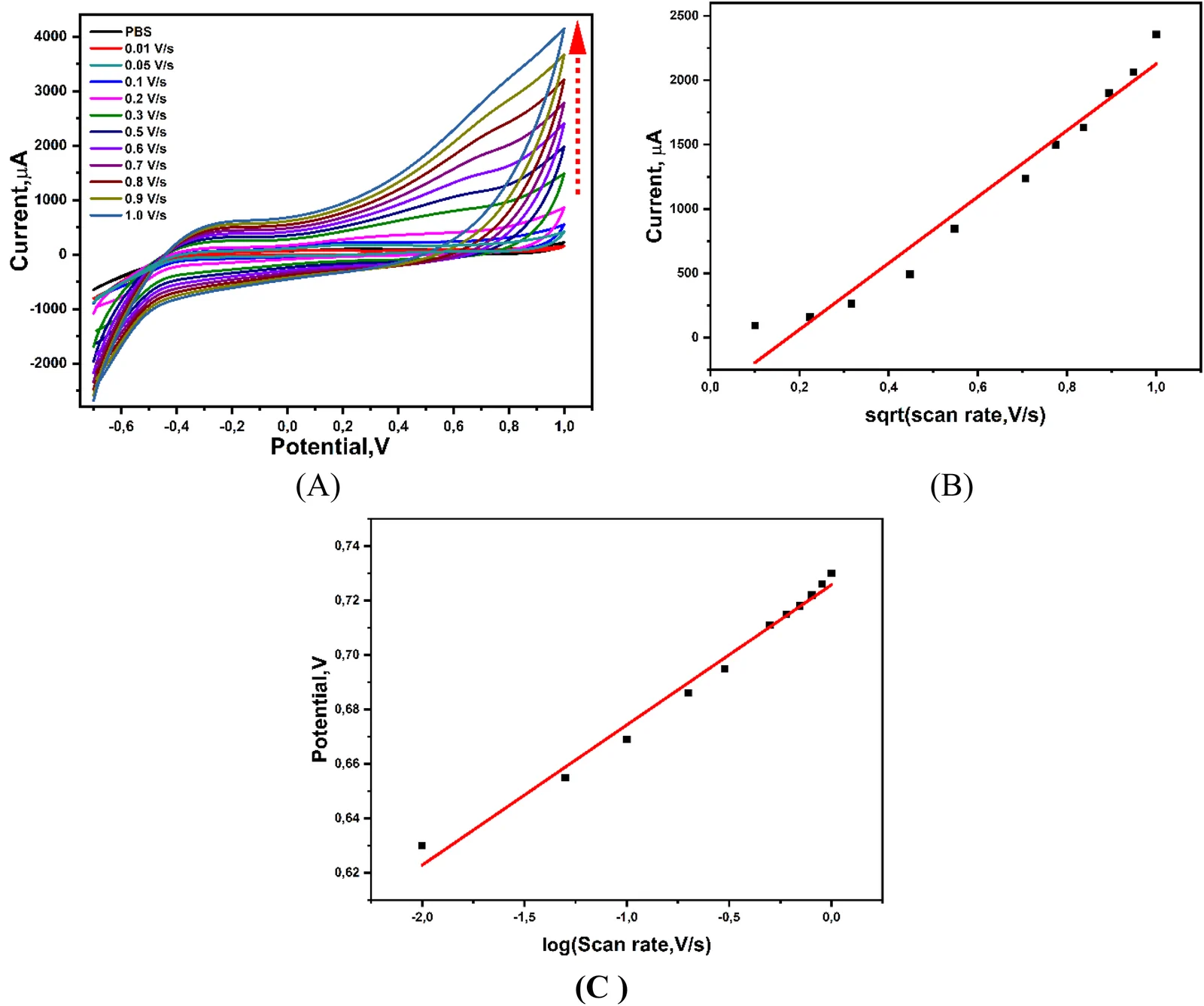
Effect of scan rate on the redox reaction(s) of hydrogen peroxide at the modified electrode surface. (A) Cyclic voltammograms recorded at different scan rates (50–1000 mV s^−1^); (B) linear relationship between anodic peak current (*I*_pa_) and the square root of scan rate (*v*^1/2^), demonstrating diffusion-controlled behavior; and (C) linear plot of anodic peak potential (*E*_p_) *versus* log *v*, used for evaluating the irreversible electron-transfer kinetics and estimating the number of transferred electrons using the Laviron model.

The anodic peak potential (*E*_p_) also shifted toward more positive potentials with increasing scan rate, indicating increased polarization and supporting the irreversible the redox process. The plot of *E*_p_*versus* log *v* (Fig. 6C) exhibited a linear relationship, with a slope of 0.0514 V decade^−1^ (*R*^2^ = 0.992). For an irreversible electrode process, the slope can be described by the Laviron relationship:*E*_p_ = *E*^0^ + (2.303*RT*/*αn* × *F*) log *v* + constantwhere *α* is the electron–transfer coefficient, *n* is the number of electrons involved in the rate-determining electrochemical step, *R* is the gas constant, *T* is the absolute temperature, and *F* is the Faraday constant. Thus, the slope (*b*) is related to the apparent *αn* product according to: *b* = 2.303*RT*/*αnF*. Using the experimentally determined slope of 0.0514 V decade^−1^ at 298.15 K, the corresponding apparent *αn* value was calculated as: *αn* = 2.303*RT*/*bF* ≈ 1.15.

This value represents the product of the electron–transfer coefficient and the number of electrons involved in the rate-determining electrochemical step. Thus, the linear dependence of *I*_pa_ on *v*^1/2^, together with the scan-rate-dependent shift of *E*_p_, indicates a substantial diffusion contribution to the observed current response accompanied by irreversible electron-transfer behavior. The overall stoichiometric oxidation of H_2_O_2_ can be represented stoichiometrically by the following two-electron reaction:H_2_O_2_ → O_2_ + 2H^+^ + 2e^−^

The observed electrochemical response can further be considered in relation to the experimentally supported contributions of the composite constituents. MnO_2_ represents the principal electrochemically active oxide component and may participate in interfacial electron–transfer processes associated with H_2_O_2_ oxidation. Previous studies have reported manganese-oxide redox transformations during H_2_O_2_ electrochemistry; therefore, Mn^4+^/Mn^3+^ transformation may represent a plausible contribution to the observed response, although this pathway was not directly verified under the present experimental conditions.^27,28^ SeO_2_ may additionally influence the interfacial electrochemical properties of the mixed-oxide phase, consistent with the comparative H_2_O_2_ responses presented in Section 3.4. The interconnected MWCNTs network provides conductive pathways that facilitate charge transport between the electroactive components and the carbon paste electrode, whereas MCC primarily serves as a supporting structural matrix contributing to the dispersion and stabilization of the composite constituents and the integrity of the modified electrode layer (Table 2).

**Table 2:** Determination of H_2_O_2_ in spiked food samples using the MCC/MnO_2_–SeO_2_@MWCNTs-modified electrode

Samples	Found (mM) real sample (*A*)	Added (mM) (spiked) (*B*)	Total found (mM) real sample + spiked (*C*)	Recovery (%) ± SD (*n* = 5) recovery = C/(*A* + *B*) × 100
Yellow Cheese	0.011	0.3	0.315	104.61 ± 1.2
		0.6	0.595	99.06 ± 1.1
		1.0	0.995	99.30 ± 1.1
Salami	0.0216	0.1	0.115	95.3 ± 2.4
		0.3	0.308	95.86 ± 3.1
		0.6	0.602	96.84 ± 3.4
		1.0	0.993	97.25 ± 2.7
Beef	0.0149	0.1	0.116	101.7 ± 1.3
		0.3	0.318	101.2 ± 1.5
		0.6	0.608	99.02 ± 1.6
		1.0	1.015	103.84 ± 0.5
Milk	0.024	0.1	0.105	103.32 ± 1.1
		0.3	0.302	100.03 ± 1.3
		0.6	0.610	101.41 ± 1.0
		1.0	1.003	100.10 ± 0.8
Burger	0.1402	0.1	0.235	97.91 ± 2.4
		0.3	0.443	100.63 ± 1.1
		0.6	0.746	100.83 ± 1.2
		1.0	1.114	97.71 ± 2.3

Accordingly, the observed H_2_O_2_ response is associated with the combined structural and electrochemical contributions of the composite constituents, which provide an accessible electrochemical interface and facilitate charge transport and interaction of H_2_O_2_ with the electroactive components. These characteristics contribute to the observed anodic response of the MCC/MnO_2_–SeO_2_@MWCNTs-CPE and support its application as a non-enzymatic electrochemical platform for H_2_O_2_ determination (Scheme 1).

**Scheme 1:**
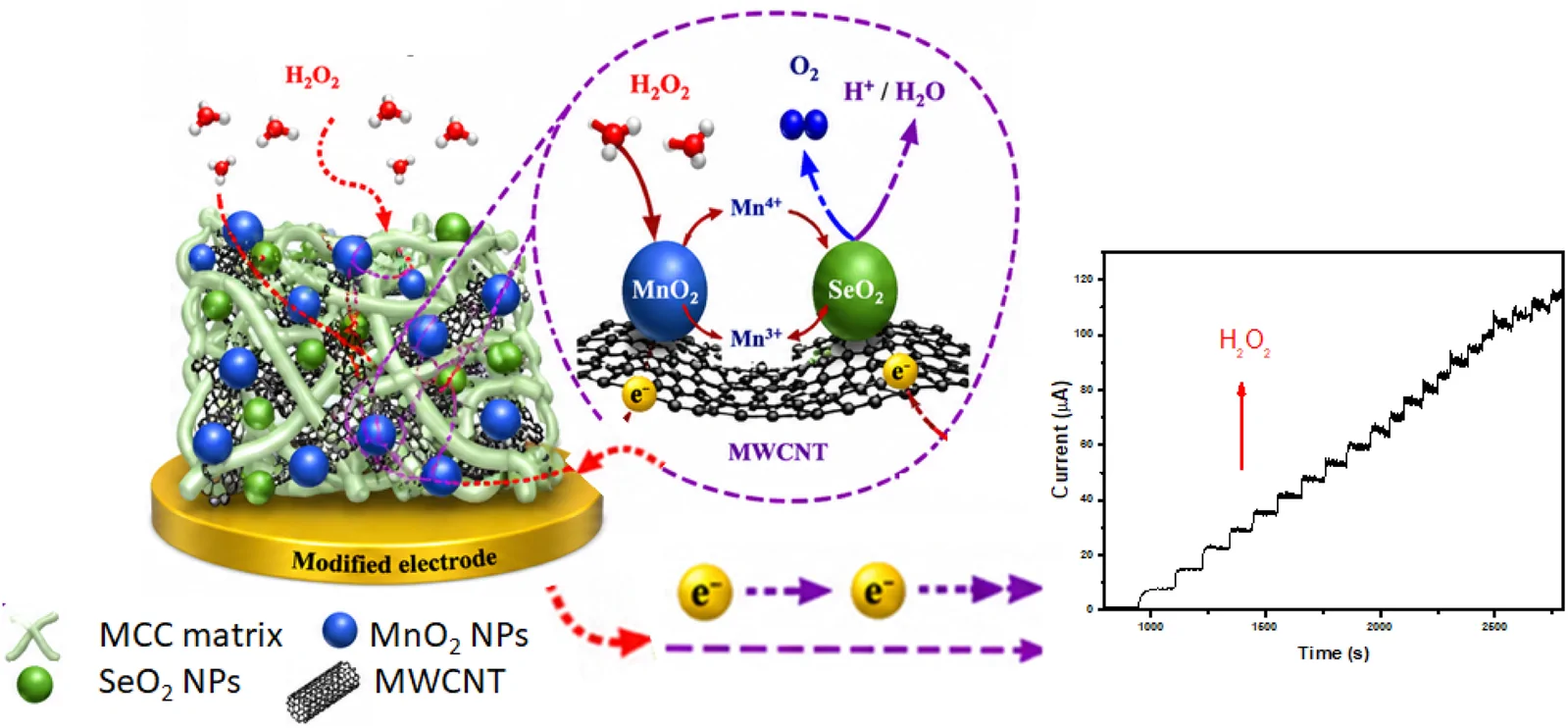
Proposed mechanism of H_2_O_2_ electrochemical oxidation at the MCC/MnO_2_–SeO_2_@MWCNTs-modified electrode.

### 3.7 Interference study, reproducibility, and electrode stability

The interference behavior of the newly developed non-enzymatic electrochemical sensor toward H_2_O_2_ was evaluated by chronoamperometry in the presence of selected potentially interfering species (Fig. 7A). A stable baseline current was first established, followed by the addition of 0.01 mM H_2_O_2_, which produced a distinct increase in the oxidation current. Subsequently, d-glucose, citric acid, sucrose, KCl, NaCl, CuSO_4_, Pb(NO_3_)_2_, ascorbic acid, CuCl_2_, and NH_4_Cl were sequentially introduced at 0.02 mM, corresponding to a two-fold excess relative to the H_2_O_2_ concentration. These species were selected to represent common food-matrix constituents, inorganic salts, electroactive compounds, and potentially interfering ionic species. The two-fold excess was deliberately selected as a defined challenge condition to evaluate the extent to which these representative species could alter the measured H_2_O_2_ response.

**Fig. 7:**
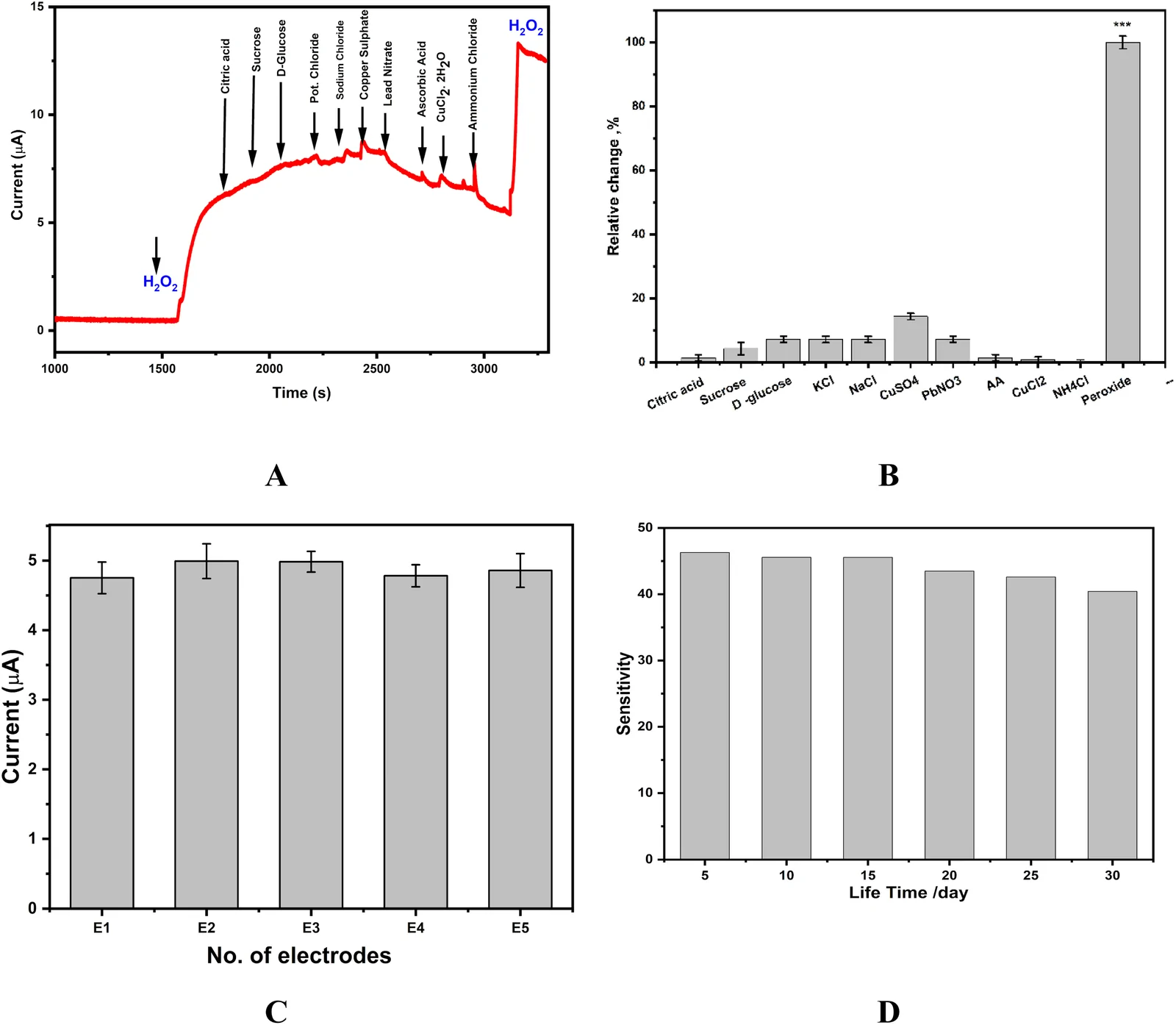
(A) CA Interference study; (B) relative change % in the presence of various interferences using the MCC/MnO_2_–SeO_2_@MWCNTs-modified electrode toward the direct oxidation of H_2_O_2_ in PBS (pH 7.0). (C) Reproducibility of the modified electrodes for the H_2_O_2_ detection using five independently prepared electrodes. (D) Storage stability of the electrodes toward H_2_O_2_ over 30 days.

The relative change in current produced by each interferent was calculated according to:Relative change (%) = *I*_H_2_O_2_+interferent_ − *I*_H_2_O_2__/*I*_H_2_O_2__ × 100where (*I*_H_2_O_2__) is the oxidation current measured after addition of H_2_O_2_ alone and (*I*_H_2_O_2_+interferent_) is the corresponding current measured after addition of the interferent. The investigated species produced relatively small changes (Fig. 7B) in the measured current. Importantly, a subsequent addition of 0.01 mM H_2_O_2_ produced a pronounced increase in the current response, confirming that the sensor retained a measurable response to H_2_O_2_ in the presence of the tested interferents.

The reproducibility of the sensor fabrication was evaluated using five independently prepared MCC/MnO_2_–SeO_2_@MWCNTs-modified CPEs under identical experimental conditions (Fig. 7C). The electrodes exhibited comparable current responses toward the same H_2_O_2_ concentration, with only a small variation among the independently prepared electrodes, indicating good electrode-to-electrode reproducibility and consistency of the fabrication procedure.

The storage stability of the modified electrodes was further investigated over a period of 30 days (Fig. 7D). The sensor maintained a relatively stable response throughout the investigated storage period, indicating satisfactory stability under the applied storage conditions. Overall, the interference, reproducibility, and storage-stability results demonstrate that the MCC/MnO_2_–SeO_2_@MWCNTs-modified electrode provides a consistent H_2_O_2_ response and retains its analytical response toward the target analyte under the investigated experimental conditions.

### 3.8 Application to real food samples

The practical applicability of the modified electrode for H_2_O_2_ determination was demonstrated using commercial yellow cheese, salami, beef, milk, and burger samples as representative food matrices. Known concentrations of H_2_O_2_ were spiked into the prepared samples, and the resulting concentrations were determined under the optimized electrochemical conditions using the established calibration relationship. The analytical accuracy was calculated from the percentage recovery.

As summarized in Table 1, the recoveries ranged from 94.57% to 104.62%, with relative standard deviations (RSDs) of 0.5–3.4% (*n* = 5). The obtained recoveries demonstrate good agreement between the measured and theoretical H_2_O_2_ concentrations across the investigated food matrices, indicating satisfactory analytical accuracy and reproducibility. The consistent performance across the different sample matrices further supports the applicability of the modified electrode for H_2_O_2_ determination in complex food samples under the employed sample-preparation and measurement conditions.

## 4. Conclusion

A hierarchical MCC/MnO_2_–SeO_2_@MWCNTs nanocomposite was developed as a non-enzymatic electrochemical sensing interface for H_2_O_2_ direct determination in food matrices. The scientific contribution of this work lies in establishing a cellulose-based hierarchical electrode architecture in which the MCC matrix is integrated with the MnO_2_–SeO_2_ phase and conductive MWCNT network to produce an electrochemically accessible sensing interface. This structural design was supported by FTIR and electron-microscopic characterization, while electrochemical measurements showed a 63% increase in electrochemically active surface area, from 0.253 to 0.412 cm^2^, following electrode modification. Under the optimized chronoamperometric conditions, the resulting electrode exhibited a linear H_2_O_2_ response over 10–1200 µM with a 1.0 µM detection limit. The sensor further demonstrated satisfactory selectivity, repeatability, reproducibility, and stability under the investigated conditions. Most importantly, its practical applicability was verified using yellow cheese, salami, beef, milk, and burger samples, providing recoveries of 95.3–104.61% (*n* = 5). These results demonstrate that the developed MCC/MnO_2_–SeO_2_@MWCNTs interface can support quantitative H_2_O_2_ determination across chemically diverse food matrices without relying on an enzymatic recognition element. Thus, this work adds the cellulose-supported, mixed-oxide/carbon nanocomposite strategy to the field of non-enzymatic H_2_O_2_ sensing and links the material architecture to a measurable increase in electrochemical accessibility and validated analytical performance in real food matrices. The demonstrated combination of a 63% ECSA enhancement, micromolar detection capability, and successful application to five food matrices provides the principal scientific and practical value of the study. This developed platform shows potential for rapid, direct, and reliable H_2_O_2_ determination in food matrices, supporting its future application in on-site food-safety monitoring following validation against established reference methods.

## Conflicts of interest

The authors declare that the research was conducted in the absence of any commercial or financial relationships that could be construed as a potential conflict of interest.

## Supplementary Material

RA-016-D6RA07921K-s001

## Data Availability

Data are available upon request from the corresponding author of this article. Supplementary information (SI): the electrochemical data analysis presented in Table S1, including the parameters extracted from the generated cyclic voltammograms and Nyquist plots obtained from the EIS measurements shown in Fig. 2B. See DOI: https://doi.org/10.1039/d6ra07921k.
