# Supplementary material for: Hierarchical microcrystalline cellulose/SeO2–MnO2/MWCNTs nanocomposite-based non-enzymatic electrochemical sensor for peroxide analysis in food and milk samples
Source: RSC Adv. 2026 Oct 8;16(47):61806–20. doi: 10.1039/d6ra07921k (PMC13648327; doi:10.1039/d6ra07921k)
Supplement: Supplementary file 1 [file RA-016-D6RA07921K-s001.pdf]

Supplementary Information (SI) for RSC Advances.
This journal is © The Royal Society of Chemistry

Supplementary materials

Hierarchical Microcrystalline Cellulose/SeO_2-MnO_2/MWCNTs Nanocomposite-Based Non-Enzymatic Electrochemical Sensor for Peroxide Analysis in Food and Milk Samples

Sara ZA Mahdy[1], Hend S. Magar[2], Rabeay Y. A. Hassan[1*]

[1]Biosensors Research Lab, Zewail City of Science and Technology, 6th October City, Giza, 12578, Egypt; [2]Applied Organic Chemistry Department, National Research Centre (NRC), Dokki, Giza 12622, Egypt

***CORRESPONDING AUTHOR**

Rabeay Y. A. Hassan, PhD

Biosensors Research Lab,

Zewail City of Science and Technology,

6th October City, Giza 12578, Egypt

Email: ryounes@zewailcity.edu.eg

Phone: +201129216152

Table S1: Electrochemical data analysis extracted from the generated cyclic voltammograms and Nyquist plots of the EIS measurements (**Figure 2B**).

Material	$I_{Ox.}$ (μA)	$I_{Red.}$ (μA)	$E_{oxd.}$ (V)	$E_{Red,}$ (V)	Rs Ω	Rct Ω	CPE n F	W Ω
Bare	188.30	-191.22	0.301	0.0888	60	290	0.000141 0.78	68.92
MCC	303.95	-302.40	0.225	0.0378	79	75	0.000172 0.85	92.45
MCC/MnO_2	240.45	-235.26	0.2511	0.1317	87	100	0.000162 0.69	84.61
MnO_2/MWCNTS	149.44	-130.84	0.405	-0.051	64	211	0.000157 0.81	79.94
MCC/MnO_2/MWCNTs	265.09	-263.09	0.318	0.0714	41	137	0.000160 0.72	83.14
MCC/MnO_2-SeO_2 @ MWCNTs	318.27	-369.55	0.261	0.1195	48	55	0.000168 0.78	88.34
